# Morphogenesis and cytopathic effect of SARS-CoV-2 infection in human airway epithelial cells

**DOI:** 10.1038/s41467-020-17796-z

**Published:** 2020-08-06

**Authors:** Na Zhu, Wenling Wang, Zhidong Liu, Chaoyang Liang, Wen Wang, Fei Ye, Baoying Huang, Li Zhao, Huijuan Wang, Weimin Zhou, Yao Deng, Longfei Mao, Chongyu Su, Guangliang Qiang, Taijiao Jiang, Jincun Zhao, Guizhen Wu, Jingdong Song, Wenjie Tan

**Affiliations:** 1 NHC Key Laboratory of Biosafety, National Institute for Viral Disease Control and Prevention, Chinese Center for Disease Control and Prevention, 102206 Beijing, China; 2 Department of Thoracic Surgery, Beijing Chest Hospital, Capital Medical University (Beijing Tuberculosis and Thoracic Tumor Research Institute), 101149 Beijing, China; 3 Department of Thoracic Surgery, China–Japan Friendship Hospital, Yinghua East Road No. 2, Chaoyang District, 100029 Beijing, China; 4 Suzhou Institute of Systems Medicine, 215123 Suzhou, Jiangsu China; 5 State Key Laboratory of Respiratory Disease, National Clinical Research Center for Respiratory Disease, Guangzhou Institute of Respiratory Health, The First Affiliated Hospital of Guangzhou Medical University, 510120 Guangzhou, China; 6 Institute of Infectious Disease, Guangzhou Eighth People’s Hospital of Guangzhou Medical University, 510120 Guangzhou, China; 7 State Key Laboratory of Infectious Disease Prevention and Control, National Institute for Viral Disease Control and Prevention, Chinese Center for Disease Control and Prevention, 102206 Beijing, China; 8 Center for Biosafety Mega-Science, Chinese Academy of Sciences, 430071 Wuhan, China

**Keywords:** SARS-CoV-2, Viral infection

## Abstract

SARS-CoV-2, a β-coronavirus, has rapidly spread across the world, highlighting its high transmissibility, but the underlying morphogenesis and pathogenesis remain poorly understood. Here, we characterize the replication dynamics, cell tropism and morphogenesis of SARS-CoV-2 in organotypic human airway epithelial (HAE) cultures. SARS-CoV-2 replicates efficiently and infects both ciliated and secretory cells in HAE cultures. In comparison, HCoV-NL63 replicates to lower titers and is only detected in ciliated cells. SARS-CoV-2 shows a similar morphogenetic process as other coronaviruses but causes plaque-like cytopathic effects in HAE cultures. Cell fusion, apoptosis, destruction of epithelium integrity, cilium shrinking and beaded changes are observed in the plaque regions. Taken together, our results provide important insights into SARS-CoV-2 cell tropism, replication and morphogenesis.

## Abstract (web summary)

Here, the authors characterize replication of SARS-CoV-2 in human airway epithelial (HAE) cultures and show that it can infect ciliated and secretory cells, affects transepithelial electrical resistance and causes plaque-like cytopathic effects associated with apoptosis.

## Introduction

A large outbreak of unusual viral pneumonia (coronavirus disease 2019, COVID-19) was first reported in Wuhan, China, in late December 2019. The rapid spread of this virus across the world in 6 months with over 10 million confirmed cases has resulted in 517,877 deaths as of July 4, 2020. A novel human β-coronavirus, named SARS-CoV-2, has been identified as the etiologic agent^1^. Although genomics characterization^2,3^ and epidemiological retrospective investigations of SARS-CoV-2^4^ have been conducted, few detailed pathology studies^5,6^ have been published due to the limited access to autopsy and biopsy specimens. The underlying pathogenesis and transmission of viral infection remain obscure.

Organotypic cell culture of well-differentiated human airway epithelial cells (HAE) has been successfully used to isolate SARS-CoV-2 and offers some advantages in sustaining SARS-CoV-2 replication compared with standard immortalized cells (such as Vero E6 or Huh7 cells)^1^. HAE cells also serve as an in vitro physiological model of human lung origin to investigate the morphogenesis and pathogenesis of SARS-CoV-2^7^. Although SARS-CoV-2, SARS-CoV, and HCoV-NL63 use the same receptor ACE2^8–11^, SARS-CoV-2 seems to spread much more efficiently simply based on the numbers of cases and transmissibility across the world in such a short time. In this study, we compared the characteristics of the replication dynamics, cell tropism, and morphogenesis of SARS-CoV-2 and HCoV-NL63 in HAE cells, which express the shared receptor, to better understand the pathogenesis and transmission of SARS-CoV-2.

## Results

### Replication dynamics of SARS-CoV-2 in human airway epithelium

**Fig. 1:**
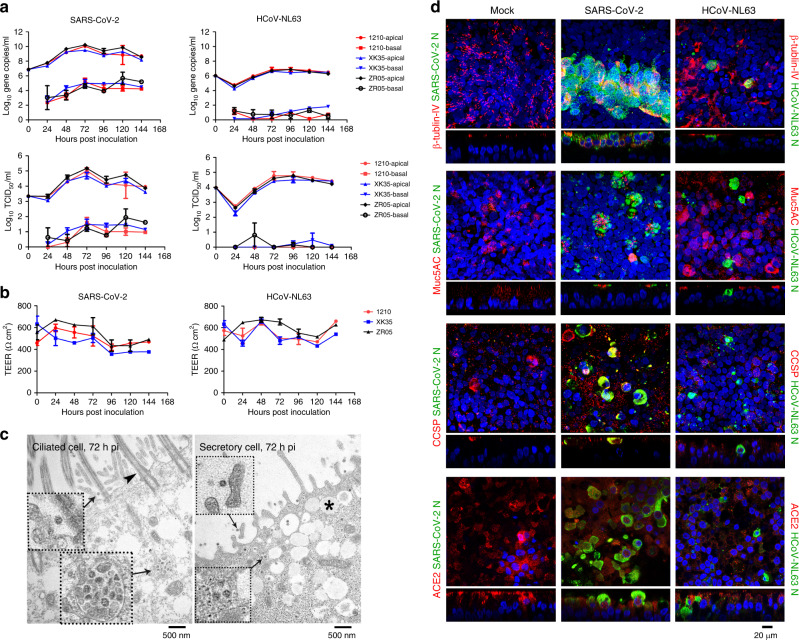
Characterization and cell tropism of SARS-CoV-2 in human airway epithelia (HAE). **a** SARS-CoV-2 replication kinetics in HAE from different donors, HCoV-NL63 was used as a control (*n* = 3). **b** Transepithelial electrical resistance (TEER in Ω cm^2^) between the apical and basal poles was measured at each time point (*n* = 3). **c** SARS-CoV-2 infected both ciliated cells (72 h pi) and secretory cells (72 h pi). arrows: virus particles, arrowhead: cilium, asterisk: secretory vesicle, insets dashed-line squares indicate magnification of arrowed areas. **d** Costaining of SARS-CoV-2 N protein (green) with ciliated cell marker β-tubulin-IV (red), goblet cell marker Muc5AC (red), club cell marker CCSP (red), and ACE2 (red) positive cells. HCoV-NL63 N protein (green) staining was used as a control (72 h pi). Nuclei were stained with 4’,6-diamidino-2-phenylindole (DAPI) (blue). Data **a**, **b** are the means ± s.d. of three independent biological replicates. Source data **a**–**d** are provided as a Source Data file.

To confirm the replication dynamics of SARS-CoV-2 in HAE, fully differentiated HAE cultures derived from three different donors (1210, XK35, ZR05) were inoculated with SARS-CoV-2 or human coronavirus NL63 (HCoV-NL63) (Amsterdam, ATCC) at a multiplicity of infection (MOI) of 0.1, which was consistent with the infection of HAE by other coronaviruses, including HCoV-NL63^12,13^, SARS-CoV^14^, MERS-CoV^15^, HCoV-229E^13,16^, HCoV-OC43^13,17^, and HCoV-HKU1^13,18^, to assess the viral growth kinetics. Additionally, the replication efficiency of the two kinds of viruses could be compared by using the same MOI to inoculate HAE cells. As shown in Fig. 1a, HAE cells were highly susceptible to SARS-CoV-2 infection with peak virus production from apical wash at 48–72 h post infection (h pi) and remained at a high level from 3 to 6 days. In contrast, HCoV-NL63 reached peak virus load at 72–96 h pi, similar to previously reported SARS-CoV replication kinetics in HAE^14^. SARS-CoV-2 progeny viruses were released into the basolateral medium as the infection progressed, yet nearly no progeny viruses were released into the basolateral medium post infection with HCoV-NL63 (Fig. 1a). We further monitored transepithelial electrical resistance (TEER), considered a surrogate of epithelium integrity, during infection with SARS-CoV-2 or HCoV-NL63, and we found that at 96 h pi, the TEER of HAE inoculated with SARS-CoV-2 was reduced nearly 40% but not in HCoV-NL63-infected cells (Fig. 1b). Notably, the decrease in TEER in SARS-CoV-2-infected HAE was accompanied by an increase in SARS-CoV-2 progeny virus detected in the basolateral medium (Fig. 1a).

### Cell tropism of SARS-CoV-2 in human airway epithelium

Previous studies showed that SARS-CoV-2 utilized ACE2 as its cell surface receptor^8,9^, suggesting that SARS-CoV-2 may share a similar cell tropism (ciliated cells in HAE) with SARS-CoV and HCoV-NL63 by using the same receptor^10,11^. To further confirm the tropism of SARS-CoV-2 in HAE, transmission electron microscopy (TEM) and laser scanning confocal microscopy analyses were performed. Surprisingly, we found that SARS-CoV-2 infects both ciliated cells and secretory cells. As shown in Fig. 1c, virus particles were found on the apical surface of both ciliated cells and secretory cells; inclusion bodies formed by viral components were observed in the cytoplasm, which confirmed the infection of both cell types. Immunofluorescent staining of SARS-CoV-2 N protein colocalized with ciliated cells (marker: β-tubulin IV) and secretory cells, including goblet cells (marker: Muc5AC) and club cells (marker: CCSP) (Fig. 1d). This was dramatically different from six other human coronaviruses. It had been demonstrated that HCoV-HKU1, HCoV-OC43, HCoV-NL63, and SARS-CoV infect ciliated cells^12,13,18,19^, while HCoV-229E and MERS-CoV infect secretory cells^13,15,16^. Additionally, the presence of SARS-CoV-2-infected ACE2-positive cells further confirmed ACE2 as a surface receptor of SARS-CoV-2 (Fig. 1d).

### Cytopathic effects (CPE) and ultrastructural pathology induced by SARS-CoV-2 in human airway epithelium

**Fig. 2:**
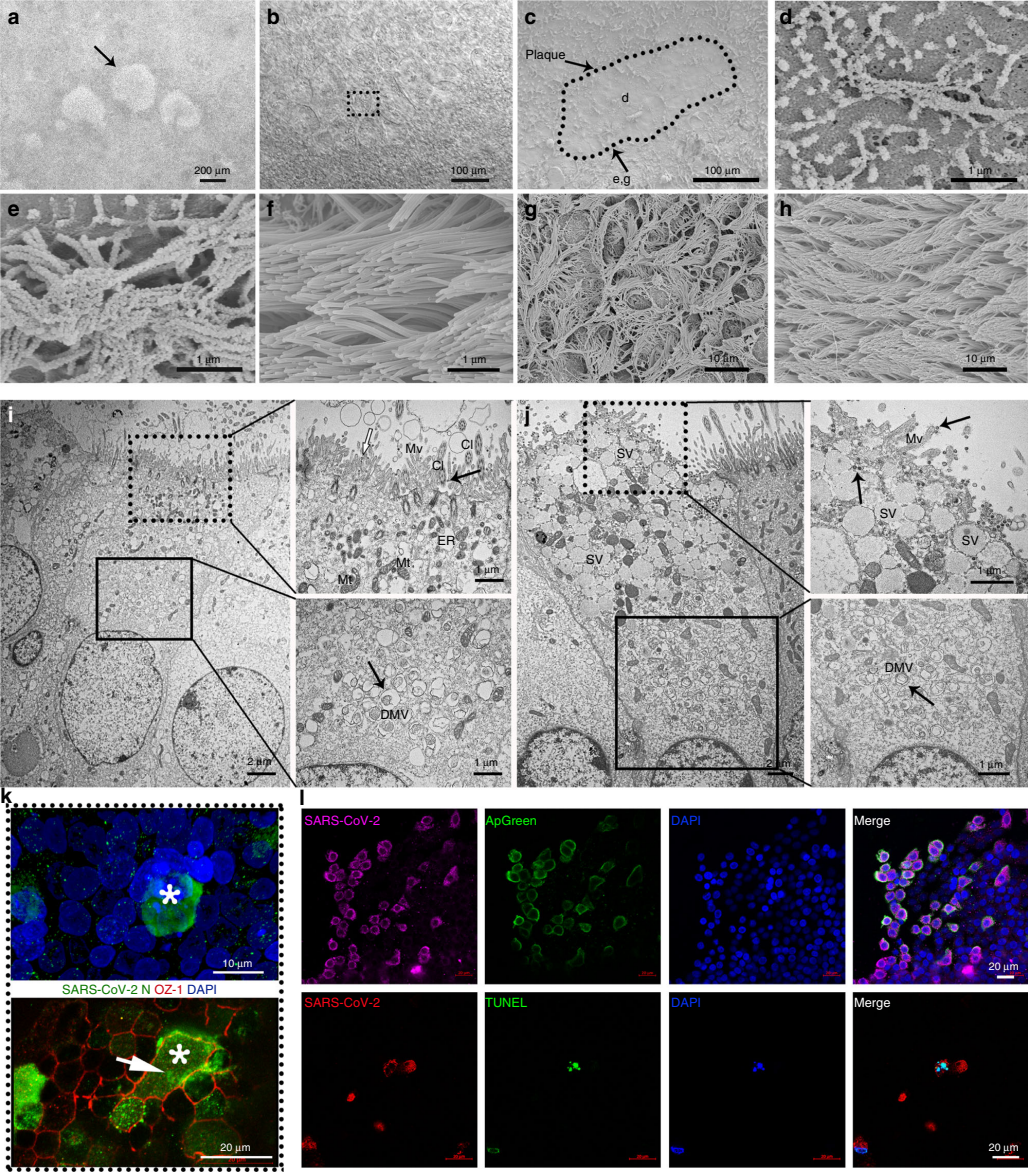
Cytopathic effect of SARS-CoV-2 infection on HAE cells. Characterization of SARS-CoV-2 infection of the HAE surface **a**–**h**. **a** Plaques (arrow) induced by SARS-CoV-2 infection under a light microscope. **b** Cell fusion and net-like structure (dashed line square) under a laser scan confocal microscope in the plaque region. **c** Plaque area featured with less content under a scanning electron microscope (dashed line circle). **d** Deformation of cilia in the plaque area under a scanning electron microscope. **e** Cilia polarity disorder and granular formation on cilium with rough surface under a scanning electron microscope. **f** Normal HAE cell cilium with polarity order. **g** Cilia polarity disorder under a scanning electron microscope. **h** Mock HAE cell cilium with smooth surface and polarized order. Ultra-pathology of SARS-CoV-2-infected HAE cells **i**, **j**. **i** Overview of a virus-infected ciliated cell. The black line box indicates double membrane vesicles (DMVs) induced by virus infection in ciliated cells. The dashed line box indicates aggregation of denatured mitochondria (Mt) and enlarged endoplasmic reticulum (ER) on the top area of ciliated cells. Virus particles on cilia (Cl) (arrow) and microvilli (Mv) (empty arrow). **j** Overview of a virus-infected secretory cell with cell organelles and secretory vesicles (SV) aggregated on the top area of the cell. The black line box indicates double membrane vesicles (DMVs) induced by virus infection in secretory cells. The dashed line box indicates virus particles both in the cytoplasm and on microvilli (Mv) (arrows). **k** Syncytial cell formation (star) and cell tight junction destruction (white arrow) caused by SARS-CoV-2 infection. **l** Apoptosis induced by SARS-CoV-2 infection in HAE. Apoptotic cells (green) stained with Apopxin Green (ApGreen) and TdT-mediated dUTP Nick-End Labeling (TUNEL) indicator. Source data **a**–**i** are provided as a Source Data file.

In our previous study, unique CPE were observed in HAE induced by SARS-CoV-2 infection^1^. To investigate the CPE in more detail, SARS-CoV-2-infected HAE were analyzed by laser scanning confocal microscopy, scanning electron microscopy (SEM) and immunofluorescence staining. Plaque-like CPE was consistently observed in different propagations of SARS-CoV-2-infected HAE (Fig. 2a). The size and number of plaques increased with the extension of the incubation time. As shown in Fig. 2b, multinucleated syncytial cells arranged in a net-like structure were observed in the plaque regions. Immunofluorescence staining using a specific SARS-CoV-2 N protein antibody and cell tight junction antibody showed giant syncytial cell formation and destruction of cell tight junctions (Fig. 2k). Cilium shrinking (Fig. 2d) in the plaque region (Fig. 2c) and beaded changes (Fig. 2e) in the periphery of plaques were detected compared with mock-infected cells (Fig. 2f). Cilia were disordered (Fig. 2g) in virus-infected cells compared with mock-infected cells (Fig. 2h) in the far periphery of the plaques.

HAE ultrathin sections were analyzed by TEM after SARS-CoV-2 infection. One of the most striking features of ultrastructural pathology was the formation of numerous pleomorphic double-membrane vesicles (DMVs) in the cytoplasm in both ciliated cells (Fig. 2i) and secretory cells (Fig. 2j). The DMVs were generally spherical and had an electron density matrix similar to that observed in the cytoplasm (Fig. 2i, j). The DMVs were similar to those caused by other coronavirus infections^20–23^. Another interesting ultrastructural alteration was the aggregation of organelles close to the apical surface, including mitochondria, vesicles and virus particles (Fig. 2i, j), viral inclusion bodies, etc. Virus particles were often present on the outer cell surface, specifically on microvilli and on cilia (i), which might be associated with their malfunction.

To further investigate the extensive cell death caused by apoptosis or necrosis in the CPE region, specific staining with a cell apoptosis/necrosis detection kit (blue, green, red) (Abcam 176749) that can detect both apoptosis and necrosis at the same time was performed. As shown in Fig. 2l, many apoptotic cells were observed in the SARS-CoV-2-infected culture, but almost no necrotic cells were detected, indicating that SARS-CoV-2 mostly induced HAE cell apoptosis but not necrosis. A One Step TUNEL Apoptosis Assay (Beyotime Biotechnology C1086) was performed to detect 180–200 bp DNA fragments, further confirming the apoptosis induced by SARS-CoV-2 infection.

### Morphogenesis of SARS-CoV-2 in ciliated cells and secretory cells

**Fig. 3:**
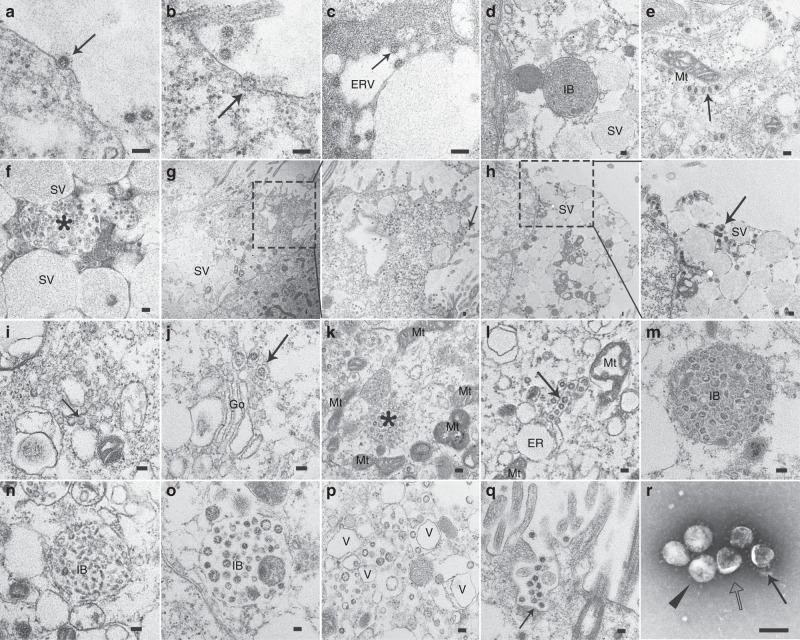
SARS-CoV-2 morphogenesis in HAE cells. SARS-CoV-2 infected both secretory cells **a**–**h** and ciliated cells **k**–**q** and presented similar morphogenetic processes (72 h p.i.). **a** Virus attaching (arrow) on the cell surface. **b** Virus and cell membrane fusion (arrow). **c** Virus budding (arrow) into endoplasmic reticulum vesicles (ERV). **d** Virus-associated inclusion bodies (IB) filled with electron condensed matrix in the cytoplasm. **e** Strands of the endoplasmic reticulum contained rows of viral particles in the cytoplasm (arrow). **f** Inclusion full of virus particles (star) with membrane-bound compressed by secretory vesicles (SV). **g** Virus particles (arrow) released from the cell together with cytoplasmic components (dashed box). **h** Virus particles (arrow) released with secretory vesicles (SV, dashed line box). **i** Provirus particles (arrow) generated in membrane-rich areas in the cytoplasm of ciliated cells. **j** Virus-containing vacuoles (arrow) in the Golgi compartments (Go). **k** Virus particle aggregation (star) with matrix but without bound membrane enclosed by mitochondria (Mt) in the cytoplasm. **l** Strands of endoplasmic reticulum containing rows of viral particles in the cytoplasm. **m** Inclusion body (IB) filled with different sizes of spherical virus particles and condensed granular matrix. **n** Inclusion body (IB) filled with pleomorphic virus particles. **o** Inclusion body (IB) filled with spherical mature virus particles of different sizes. **p** Scattered virus particles in the vesicle (V)-rich area in the cytoplasm. **q** Virus particle release by exocytosis (arrow). **r** The negatively stained SARS-CoV-2 are spherical. Particle with distinctive spikes (arrow), without spikes (empty arrow) or with partial spikes (triangle). Scale bar: 100 nm. Source data **a**–**r** are provided as a Source Data file.

To investigate SARS-CoV-2 morphogenesis in HAE cells, ultrathin sections were prepared and observed by TEM. Different stages of the SARS-CoV-2 life cycle in secretory cells were detected (Fig. 3a–h). Virus infection was initiated by attaching to the cytoplasmic membrane (Fig. 3a), and virus–cell membrane fusion occurred (Fig. 3b). Nascent particles were formed by budding of viral nucleocapsids along cytoplasmic membranes into vesicles (Fig. 3c). Inclusion bodies associated with virus infection presented in the cytoplasm and contained membrane-bound, condensed matrix-associated virus-like particles (Fig. 3d). In the cytoplasm, strands of the endoplasmic reticulum containing rows of viral particles were found (Fig. 3e). Virus particles were observed in membrane-bound vesicles, either as single particles or as groups in enlarged vesicles (Fig. 3f). Free virus particles dispersed in the cytoplasm (Fig. 3g) or between secretory vesicles (Fig. 3h) were released by the secretory cells through exocytosis. The process of viral morphogenesis in ciliated cells was similar but not identical to that in secretory cells. Provirus-like particles were observed in areas that were enriched in the cytoplasmic membrane structure (Fig. 3i). Virions also appeared in Golgi cisternae (Fig. 3j). Virus particles mixed with granular aggregates were found. These virus particles were not enclosed within a binding membrane but by mitochondria (Fig. 3k). Similar to the observations in secretory cells, strands of endoplasmic reticulum containing rows of viral particles were found next to mitochondria and enlarged endoplasmic reticulum (Fig. 3l). Various types of inclusion bodies containing virus or virus-like particles with bound membranes were observed in the cytoplasm, including (1) inclusion bodies filled with virus-like particles and dense, granular material (Fig. 3m); (2) inclusion bodies filled with pleomorphic virus-like particles (Fig. 3n); and (3) inclusion bodies filled with spherical particles of different sizes (Fig. 3o). The inclusion bodies tended to migrate toward the apical surface and fused with the plasma membrane, releasing viral particles out of host cells. Virus particles were scattered in areas enriched with vesicles in the cytoplasm (Fig. 3p). The release of virus particles from ciliated cells appeared to occur through exocytosis (Fig. 3q). In conclusion, based on our morphologic results, SARS-CoV-2 has a morphogenetic process similar to that of other coronaviruses^24–26^, except for its multicellular tropism, which includes ciliated cells and secretory cells.

To provide more morphological details on SARS-CoV-2, negative staining TEM was performed. SARS-CoV-2 viral particles released in culture supernatants exhibited spherical shaped morphology and pleomorphism with distinct surface projections of spike proteins on the envelope, which were similar to those of other members in the family *Coronaviridae*. The virions possessed an average diameter of 100 nm (range from 60 to 140 nm). Spikes varied from 12.5 to 20.4 nm in length with an average length of 17.7 nm. Virus particles with partial or no spikes were also observed in culture supernatants (Fig. 3r).

## Discussion

The organotypic HAE system forms a pseudostratified epithelial layer that morphologically and functionally resembles the human airway. The cultures of basal, ciliated, and secretory cells produced protective mucus and beating cilia that are visible under a light microscope^27^. ACE2 is mainly expressed on ciliated epithelial cells of the human lungs^8^ and is thought to be the cell surface receptor for SARS-CoV-2^10^. Interestingly, SARS-CoV and HCoV-NL63 share the same receptor as SARS-CoV-2 and a tropism for ciliated cells. Here, we reported SARS-CoV-2 infection of both ciliated cells and secretory cells in HAE, which consequently suggested the possibility of more receptors for SARS-CoV-2 attachment in addition to the ACE2 receptor^10^. We also described parts of the morphogenesis process and cytopathic effect of SARS-CoV-2 infection in both cell types in detail.

Epidemiological investigations have suggested that SARS-CoV-2 is highly infectious and transmissible among humans. In comparison to SARS-CoV or MERS-CoV, SARS-CoV-2 replicated more efficiently in primary HAE than in standard immortalized cells. This might be because there were more susceptible cells to SARS-CoV-2 infection than to other coronavirus infections. SARS-CoV-2 replicates more efficiently than HCoV-NL63 in HAE. This observation itself indicated that SARS-CoV-2 is more transmissible. The efficient transmissibility may be due to there being one more permissible cell type for SARS-CoV-2 transmission. Furthermore, the secretory cells released vesicles with large amounts of virus particles dispersed in the cytoplasm by exocytosis, which underlies the high detection rate of SARS-CoV-2 in sputum^28^ and its transmission by droplets. Additionally, few pathologies have been reported due to the limited access to autopsy or biopsy specimens, but all the reports^5,6^ have referred to the observation of a large amount of foam or gelatinous mucus in the trachea at autopsy. This may be induced by infection of secretory cells and dysregulation of mucus secretion balance, indicating that clinical treatment should be considered to restore the balance in mucus secretion.

Respiratory ciliary function abnormalities have been associated with various diseases, such as cystic fibrosis, chronic obstructive pulmonary disease, and sinusitis^29^. In this study, large areas of disordered cilia were visually confirmed by SEM analysis, indicating that SARS-CoV-2 infection disrupted cilia synchronicity. Cilium shrinking in the center of CPE plaque was first observed as no ciliary beating under the light microscope could be found. The beaded changes in cilia in the peripheral region of plaques were considered characteristic of severe pathological changes. The abnormal ciliary beating and disruption of cilia synchronicity lead to poor mucociliary clearance (MCC), which can result in secondary infections^30^. Aggregation of organelles close to the apical surface featuring a large number of mitochondria with abnormal morphology was also identified as another striking ultrastructural change. The extensive cell death observed in the CPE region was shown to be apoptosis.

In addition, unique plaque-like CPE on the apical surface of HAE were observed consistently across different propagations. The size and numbers of these plaques expanded and increased over the incubation period as well as the formation of syncytial cells and destruction of cell tight junctions. Collectively, our findings also suggested that SARS-CoV-2 might either be released via the apical surface of infected cells or transmit through direct cell-to-cell contact.

Taken together, our data support the notion that SARS-CoV-2 is fully adapted to the human airway, which is distinct from other coronaviruses that were reported to have interspecies transmission. Our study shows SARS-CoV-2 multicellular tropism and severe ultrapathological changes and malfunction of cilia, which could provide clues for clinical treatment and drug screening strategies. Recent publications^7,31^ indicated a high expression level of the SARS-CoV-2 receptor ACE2 gene in both goblet cells and ciliated cells by scRNA sequencing, which is consistent with our study. One of the limitations in our work is the lack of host response analyses after HAE are infected with SARS-CoV-2 as has been performed in a recent study^32^. Nevertheless, the results reported here should improve the understanding of SARS-CoV-2 transmission and pathogenesis.

## Methods

### Cell cultures

Human tracheobronchial epithelial cells from three different donors (1210, XK35, ZR05) were obtained from patients who underwent bronchoscopy and/or surgical lung resection during their diagnostic process for any pulmonary disease and who gave informed consent. This study was approved by the ethics committee of National Institute for Viral Disease Control and Prevention, China CDC. Resected cells from airway specimens were isolated following the method reported previously^33^ by Fulcher and were stored in liquid nitrogen until differentiation in air–liquid interface culture. Briefly, primary cells were expanded on plastic to confluency (PneumaCult^TM^-ExPlus Medium, Stemcell) and plated at a density of 250,000 cells per well on permeable Transwell-Col (12-mm-diameter) supports. HAE cultures were generated by providing an air–liquid interface (PneumaCult^TM^-ALI Medium, Stemcell) for 4–6 weeks to form well-differentiated, polarized cultures that resemble in vivo pseudostratified mucociliary epithelium. Vero E6 (CL158) and LLC-MK2 (CCL7) cells were obtained from American Type Culture Collection (ATCC, Manassas, VA) and maintained in Dulbecco’s modified Eagle’s medium (DMEM) with 10% fetal calf serum (FCS).

### Infection protocol

Prior to apical inoculation, the apical surfaces of well-differentiated human airway cell cultures (HAE) were rinsed three times with phosphate-buffered saline (PBS) at 37 °C. Apical surfaces were inoculated with SARS-CoV-2 (isolated, BetaCoV/Wuhan/IVDC-HB-01/2020|EPI_ISL_402119) or human coronavirus NL63 (Amsterdam, ATCC) at a MOI of 0.1, which was determined by both using quantitative real-time reverse transcription-PCR (qRT-PCR) specific for SARS-CoV-2 or HCoV-NL63 and titration of infectious particles on Vero E6 or LLC-MK2 cells. (Primer and probe sequences are included in Supplementary Information.) To generate growth curves at specific times (0, 24, 48, 72, 96, 120, 144 h) after viral inoculation, 200 µl of PBS was applied to the apical surface of HAE and collected after a 10-min incubation at 37 °C. All samples were stored at −80 °C until assayed for RNA quantitatively or by TCID50. All virus culture work was performed in a biological safety cabinet in a biosafety level 3 laboratory.

### Measurement of TEER

The TEER of primary HAE cells was monitored by an epithelial Volt-Ohm Meter (EVOM; WPI, Sarasota, FL). Medium was added to the apical and basolateral surfaces, and TEER was measured between electrodes. All TEER values were corrected for background from the Transwell.

### Transmission electron microscopy

For negative staining, SARS-CoV-2-infected HAE cell supernatant was collected, inactivated with 2% paraformaldehyde for at least 2 h at room temperature and ultracentrifuged to sediment virus particles. Samples were absorbed on formvar and carbon film-coated grids for 1 min and then stained with 1% (W/V) phosphotungstic acid (pH 6.8) for 1 min. Grids were air dried and ready for detection.

For ultrathin sections, SARS-CoV-2-infected and mock-infected HAE cells were scraped off the membrane of the Transwell cups. Cells were then centrifuged at 1000×*g* for 10 min to form a pellet. Cell pellets were fixed with 2% paraformaldehyde–2.5% glutaraldehyde solution for at least 4 h. Then, the cells were fixed with 1% osmium tetroxide for 1.5 h, dehydrated in gradient ethanol, embedded in epoxy resin PON812 and polymerized at 60 °C for 24 h. Ultrathin sections (80 nm thickness) were obtained from the resin blocks and were placed on copper grids and stained with uranyl acetate and lead citrate. Finally, the negatively stained grids and ultrathin sections were observed under a Tecnai12 transmission electron microscope (FEI, Eindhoven, Netherlands) at 120 kV and recorded with a CCD camera.

### Scanning electron microscopy

SARS-CoV-2-infected HAE cells and mock-infected HAE cells were fixed with 2% paraformaldehyde–2.5% glutaraldehyde solution in situ for at least 4 h, washed and dehydrated in gradient ethanol, critical point dried, and vacuum evaporated with platinum. Then, the membrane with HAE cells was cut off from the Transwell rack and glued to a sample holder, and the samples were detected under a scanning electron microscope (Hitachi SU8020, Japan).

### Laser scanning confocal microscopy

SARS-CoV-2-infected HAE cells and control cells were fixed with 4% paraformaldehyde in situ for at least 8 h, permeabilized with Triton X100, blocked with 10% BSA, reacted with mouse polyclonal anti-SARS-CoV-2 N protein antibody, rabbit monoclonal β-tubulin IV antibody, rabbit monoclonal Muc5AC antibody, rabbit polyclonal CCSP antibody, and the secondary antibody goat anti-mouse IgG (H + L) Alexa Fluor^®^488 or goat anti-rabbit IgG (H + L) Alexa Fluor^®^647 following 4′,6-diamidino-2-phenylindole (DAPI) staining. Then, the cell layer was cut from the Transwell rack and placed on a glass slide sealed with a 0.17 mm thick coverslip with the cells facing the coverslip. Samples were detected and recorded under a laser scanning confocal microscope system (Zeiss LSM 880 Ariyscan with STEDYCON, Germany).

### Antibodies list

The mouse and rabbit polyclonal Ab against SARS-CoV-2 N protein was homemade and used at a dilution of 1:500 when labeling. Purchased antibody data are shown in Table 1.

**Table 1:** Source of antibodies and dyes with work concentration for immunofluorescence.

Antibodies and dyes	Source and work concentration
ACE2	Abcam (ab15348), rabbit polyclonal, 5 µg/ml
ACE2	Bioss (bs-1004R), rabbit polyclonal (1:100)
ACE2	Sino biologicals (10108-T56), rabbit polyclona (1:100)
Tubulin-IV	Abcam (ab179504), rabbit monoclonal (1:500)
Tubulin-IV	Abcam (ab11315), mouse monoclonal, 5 µg/ml
Muc5AC	Abcam (ab178294), rabbit monoclonal (1:250)
Muc5AC	Bioss (bs-7166R), rabbit polyclonal (1:250)
CCSP	Abcam (ab40873), rabbit polyclonal (1:500)
ZO-1 TJP1	Invitrogen, (402200), rabbit polyclonal, 2.5 µg/ml
Alexa Fluor^®^ 488, 594, 647 goat anti-mouse IgG (H + L)	Life Technologies A-10680 (1:1000); A-11005 (1:1000); A-21235 (1:1000)
Alexa Fluor^®^ 488, 594, 647 goat anti-rabbit IgG (H + L)	Life Technologies A-11008 (1:1000); A-11012 (1:1000); A-21244 (1:1000)
DAPI	Abcam (ab228594), 1:1000

### Reporting summary

Further information on research design is available in the Nature Research Reporting Summary linked to this article.

## Supplementary information

Supplement Information

Peer Review File

Reporting Summary

## Data Availability

The authors declare that all data supporting the findings of this study are available within the paper and supplementary information files. The source data underlying Figs. 1–3 and Supplementary Materials are provided as a Source Data file.
