# Supplementary material for: Morphogenesis and cytopathic effect of SARS-CoV-2 infection in human airway epithelial cells
Source: Nat Commun. 2020 Aug 6;11:3910. doi: 10.1038/s41467-020-17796-z (PMC7413383; doi:10.1038/s41467-020-17796-z)
Supplement: Supplementary file 1 — Supplement Information [file 41467_2020_17796_MOESM1_ESM.pdf]

Supplementary information for

Morphogenesis and cytopathic effect of SARS-CoV-2 infection in human airway epithelial cells

Na Zhu[1,9], Wenling Wang[1,9], Zhidong Liu[2,9], Chaoyang Liang[3,9], Wen Wang[1], Fei Ye[1,], Baoying Huang[1], Li Zhao[1], Huijuan Wang[1], Weimin Zhou[1], Yao Deng[1], Longfei Mao[4], Chongyu Su[2], Guangliang Qiang[3], Taijiao Jiang[4], Jincun Zhao[5,6], Guizhen Wu[1], Jingdong Song[1,7]*, Wenjie Tan[1,8,]*.

1. NHC Key Laboratory of Biosafety, National Institute for Viral Disease Control and Prevention, China CDC, Beijing 102206, China
2. Department of Thoracic Surgery, Beijing Chest Hospital, Capital Medical University Beijing Tuberculosis and Thoracic Tumor Research Institute, Beijing, 101149, China.
3. Department of Thoracic Surgery, China–Japan Friendship Hospital, Yinghua East Road No 2, Chaoyang District, Beijing 100029, People's Republic of China
4. Suzhou Institute of Systems Medicine, Suzhou, Jiangsu 215123, China
5. State Key Laboratory of Respiratory Disease, National Clinical Research Center for Respiratory Disease, Guangzhou Institute of Respiratory Health, the First Affiliated Hospital of Guangzhou Medical University, Guangzhou, 510120, China
6. Institute of Infectious Disease, Guangzhou Eighth People's Hospital of Guangzhou Medical University, Guangzhou 510120, China
7. State Key Laboratory of Infectious Disease Prevention and Control, National Institute for Viral Disease Control and Prevention, Chinese Center for Disease Control and Prevention, Beijing 102206, China
8. Center for Biosafety Mega-Science, Chinese Academy of Sciences, Wuhan 430071, China
9. Co-first authors

*Corresponding Authors:
Jingdong Song, MD, PhD,
E-mail: songjd@ivdc.chinacdc.cn
Wenjie Tan, MD, PhD,
E-mail: tanwj@ivdc.chinacdc.cn.

Supplemental data

The conventional detection method for SARS-CoV-2 infection is real-time RT-PCR with two targets (ORF1ab, N) positive in the same specimen. Primers and probes described as below:

Target Ⅰ (ORF1ab) :

Forward primer (F) : CCCTGTGGGTTTTACACTTAA
Reverse primer (R) : ACGATTGTGCATCAGCTGA
Fluorescent probe (P) : 5 '- the FAM - CCGTCTGCGGTATGTGGAAAGGTTATGG - BHQ1-3'

Target Ⅱ (N) :

Forward primer (F) : GGGGAACTTCTCCTGCTAGAAT
Reverse primer (R) : CAGACATTTTGCTCTCAAGCTG
Fluorescent probe (P) : 5' –FAM-TTGCTGCTGCTTGACAGATT-TAMR-3
Judgment of results
Negative: no Ct value or Ct value >40.
Positive: Ct value <37 can be reported as positive.
Suspicious: the Ct value is between 37-40, it is recommended to repeat the experiment. If the Ct value is less than 40, and the amplification curve has obvious peaks, the sample is judged to be positive; otherwise, it is negative.

The conventional detection method for HCoV-NL63 infection is real-time RT-PCR with one targets (N) positive in the same specimen. Primers and probes described as below:

Forward primer (F) : AGGACCTTAAATTCAGACAACGTTCT
Reverse primer (R) : GATTACGTTTGCGATTACCAAGACT
Fluorescent probe (P) : FAM-TAACAGTTTTAGCACCTTCCTTAGCAACCCAAACA-BHQ1
