# Supplementary material for: Morphogenesis and cytopathic effect of SARS-CoV-2 infection in human airway epithelial cells
Source: Nat Commun. 2020 Aug 6;11:3910. doi: 10.1038/s41467-020-17796-z (PMC7413383; doi:10.1038/s41467-020-17796-z)
Supplement: Supplementary file 3 — Reporting Summary [file 41467_2020_17796_MOESM3_ESM.pdf]

nature research

Corresponding author(s): Jingdong Song, Wenjie Tan

Last updated by author(s): Jul 8, 2020

Reporting Summary

Nature Research wishes to improve the reproducibility of the work that we publish. This form provides structure for consistency and transparency in reporting. For further information on Nature Research policies, see our Editorial Policies and the Editorial Policy Checklist.

Statistics

For all statistical analyses, confirm that the following items are present in the figure legend, table legend, main text, or Methods section.

n/a	Confirmed	
☒	☐	The exact sample size (*n*) for each experimental group/condition, given as a discrete number and unit of measurement
☒	☐	A statement on whether measurements were taken from distinct samples or whether the same sample was measured repeatedly
☒	☐	The statistical test(s) used AND whether they are one- or two-sided<br>*Only common tests should be described solely by name; describe more complex techniques in the Methods section.*
☒	☐	A description of all covariates tested
☒	☐	A description of any assumptions or corrections, such as tests of normality and adjustment for multiple comparisons
☒	☐	A full description of the statistical parameters including central tendency (e.g. means) or other basic estimates (e.g. regression coefficient) AND variation (e.g. standard deviation) or associated estimates of uncertainty (e.g. confidence intervals)
☒	☐	For null hypothesis testing, the test statistic (e.g. *F*, *t*, *r*) with confidence intervals, effect sizes, degrees of freedom and *P* value noted<br>*Give P values as exact values whenever suitable.*
☒	☐	For Bayesian analysis, information on the choice of priors and Markov chain Monte Carlo settings
☒	☐	For hierarchical and complex designs, identification of the appropriate level for tests and full reporting of outcomes
☒	☐	Estimates of effect sizes (e.g. Cohen's *d*, Pearson's *r*), indicating how they were calculated

Our web collection on statistics for biologists contains articles on many of the points above.

Software and code

Policy information about availability of computer code

Data collection	All transmission electron microscope photographs were recorded by MORADA G3 CCD camera(EMSIS, Germany) with software RADIUS under transmission electron microscope (model Tecnai12, FEI, Eindhoven, Netherlands) at 120kv.<br>All scan electron microscope photographs were taken by model SU8020 (Hitachi, Japan)<br>All laser scan confocal microscope photographs were taken by a LSM 880 Ariyscan with STEDYCON system (LSM 880 Ariyscan with STEDYCON, ZEISS, Germany)
Data analysis	GraphPad Prism 5, Photoshop CS-6

For manuscripts utilizing custom algorithms or software that are central to the research but not yet described in published literature, software must be made available to editors and reviewers. We strongly encourage code deposition in a community repository (e.g. GitHub). See the Nature Research guidelines for submitting code & software for further information.

Data

Policy information about availability of data

All manuscripts must include a data availability statement. This statement should provide the following information, where applicable:

- Accession codes, unique identifiers, or web links for publicly available datasets
- A list of figures that have associated raw data
- A description of any restrictions on data availability

The authors declare that all data supporting the findings of this study are available within the paper and supplementary information files

Field-specific reporting

Please select the one below that is the best fit for your research. If you are not sure, read the appropriate sections before making your selection.

☒ Life sciences ☐ Behavioural & social sciences ☐ Ecological, evolutionary & environmental sciences

For a reference copy of the document with all sections, see nature.com/documents/nr-reporting-summary-flat.pdf

Life sciences study design

All studies must disclose on these points even when the disclosure is negative.

Sample size	HAE cells from three different donors were including
Data exclusions	N/A
Replication	All the expetiments were performed at least 3 times with 3 different samples. Replication.
Randomization	All the graph got randomly.
Blinding	Negative

Reporting for specific materials, systems and methods

We require information from authors about some types of materials, experimental systems and methods used in many studies. Here, indicate whether each material, system or method listed is relevant to your study. If you are not sure if a list item applies to your research, read the appropriate section before selecting a response.

Materials & experimental systems

n/a	Involved in the study	
☐	☒	Antibodies
☐	☒	Eukaryotic cell lines
☒	☐	Palaeontology and archaeology
☒	☐	Animals and other organisms
☒	☐	Human research participants
☒	☐	Clinical data
☒	☐	Dual use research of concern

Methods

n/a	Involved in the study	
☒	☐	ChIP-seq
☒	☐	Flow cytometry
☒	☐	MRI-based neuroimaging

Antibodies

Antibodies used

ACE2 Abcam (ab15348) rabbit polyclonal 5 μg/ml
ACE2 Bioss (bs-1004R) rabbit polyclonal (1:100)
ACE2 Sino biologicals (10108-T56) rabbit polyclonal (1:100)
Tubulin-IV Abcam (ab179504) rabbit monoclonal (1:500)
Tubulin-IV Abcam (ab11315) mouse monoclonal 5 μg/ml
Muc5AC Abcam (ab178294) rabbit monoclonal (1:250)
Muc5AC Bioss (bs-7166R) rabbit polyclonal (1:250)
CCSP Abcam (ab40873) rabbit polyclonal (1:500)
ZO-1 TJP1 Invitrogen (402200) rabbit polyclonal 2.5 μg/ml
Alexa Fluor® 448, 594, 647goat anti-mouse IgG (H+L) Life Technologies A-10680 (1:1000) A-11005 (1:1000) A-21235(1:1000)
Alexa Fluor® 448, 594, 647goat anti-rabbit IgG (H+L) Life Technologies A-11008 (1:1000) A-11012 (1:1000) A-21244 (1:1000)
DAPI Abcam (ab228594) 1:1000

Validation

ACE2 Abcam (ab15348) rabbit polyclonal delilvery date 15th Mar, 2020. Valid for 12 months from the date delivery
ACE2 Bioss (bs-1004R) rabbit polyclonal Valid Apr 2021
ACE2 Sino biologicals (10108-T56) rabbit polyclonal delilvery date 15th Mar, 2020. Valid for 12 months from the date delivery
Tubulin-IV Abcam (ab179504) rabbit monoclonal delilvery date 15th Mar, 2020. Valid for 12 months from the date delivery
Tubulin-IV Abcam (ab11315) mouse monoclonal delilvery date 15th Mar, 2020. Valid for 12 months from the date delivery
Muc5AC Abcam (ab178294) rabbit monoclonal delilvery date 15th Mar, 2020. Valid for 12 months from the date delivery
Muc5AC Bioss (bs-7166R) rabbit polyclonal Valid Aug 2020
CCSP Abcam (ab40873) rabbit polyclonal delilvery date 15th Mar, 2020. Valid for 12 months from the date delivery
ZO-1 TJP1 Invitrogen (402200) rabbit polyclonal delilvery date 15th Mar, 2020. Valid for 12 months from the date delivery

Eukaryotic cell lines

Policy information about cell lines

Cell line source(s)	Vero purchased from ATCC (USA)
Authentication	N/A
Mycoplasma contamination	No mycoplasma contamination
Commonly misidentified lines (See ICLAC register)	N/A
