# Supplementary material for: Morphogenesis and cytopathic effect of SARS-CoV-2 infection in human airway epithelial cells
Source: Nat Commun. 2020 Aug 6;11:3910. doi: 10.1038/s41467-020-17796-z (PMC7413383; doi:10.1038/s41467-020-17796-z)
Supplement: Supplementary file 2 — Peer Review File [file 41467_2020_17796_MOESM2_ESM.pdf]

Editorial Note: This manuscript has been previously reviewed at another journal that is not operating a transparent peer review scheme. The manuscript was considered suitable for publication without further review at *Nature Communications*.
